# Nanobiochar Associated Ammonia Emission Mitigation and Toxicity to Soil Microbial Biomass and Corn Nutrient Uptake from Farmyard Manure

**DOI:** 10.3390/plants12091740

**Published:** 2023-04-23

**Authors:** Muhammad Imtiaz Rashid, Ghulam Abbas Shah, Zahid Iqbal, Muhammad Ramzan, Mohammad Rehan, Nadeem Ali, Khurram Shahzad, Ahmad Summan, Iqbal M. I. Ismail, Gabrijel Ondrasek

**Affiliations:** 1 Center of Excellence in Environmental Studies, King Abdulaziz University, P.O. Box 80216, Jeddah 21589, Saudi Arabia; 2 Department of Agronomy, Pir Mehr Ali Shah Arid Agriculture University, Rawalpindi 46300, Pakistandr.ramzanjani2@gmail.com (M.R.); 3 Department of Soil Science, Pir Mehr Ali Shah Arid Agriculture University, Rawalpindi 46300, Pakistan; 4 Department of Environment, Faculty of Environmental Sciences, King Abdulaziz University, P.O. Box 80208, Jeddah 21589, Saudi Arabia; 5 Department of Chemistry, Faculty of Science, King Abdulaziz University, P.O. Box 80200, Jeddah 21589, Saudi Arabia; 6 Department of Soil Amelioration, Faculty of Agriculture, University of Zagreb, 10000 Zagreb, Croatia

**Keywords:** agro-nanotechnology, decomposition, fertilizer use efficiency, microbial toxicity, nutrient mineralization, nanotoxicity

## Abstract

The unique properties of NB, such as its nano-size effect and greater adsorption capacity, have the potential to mitigate ammonia (NH_3_) emission, but may also pose threats to soil life and their associated processes, which are not well understood. We studied the influence of different NB concentrations on NH_3_ emission, soil microbial biomass, nutrient mineralization, and corn nutrient uptake from farmyard manure (FM). Three different NB concentrations i.e., 12.5 (NB1), 25 (NB2), and 50% (NB3), alone and in a fertilizer mixture with FM, were applied to corn. NB1 alone increased microbial biomass in soil more than control, but other high NB concentrations did not influence these parameters. In fertilizer mixtures, NB2 and NB3 decreased NH_3_ emission by 25% and 38%, respectively, compared with FM alone. Additionally, NB3 significantly decreased microbial biomass carbon, N, and soil potassium by 34%, 36%, and 14%, respectively, compared with FM. This toxicity to soil parameters resulted in a 21% decrease in corn K uptake from FM. Hence, a high NB concentration causes toxicity to soil microbes, nutrient mineralization, and crop nutrient uptake from the FM. Therefore, this concentration-dependent toxicity of NB to soil microbes and their associated processes should be considered before endorsing NB use in agroecosystems.

## 1. Introduction

Agricultural activities are the primary cause of ammonia (NH_3_) emissions into the environment [1]. Among them, the livestock sector contributes 60% of these global emissions [2]. There is a global consensus that major portions of ammonium (NH_4_^+^) and NH_3_ found in the atmosphere come from livestock manure management, from its handling to storage and land application operations [3]. A recent global meta-analysis found that the soil application of animal manure was responsible for a 14.1% global effective NH_3_ emission rate, which was 12.4% higher than that of NH_3_ emitted from synthetic N fertilizer [4]. These emissions from animal manure reduce its N fertilizer use efficiency and crop production, leading to low crop yields and additional costs. The use of additional chemical or organic fertilizers may overcome these costs [5], but it may also cause more N loss to the environment. Such a high amount of atmospheric N leads to wet and dry deposition in soil or waterways, causing N eutrophication and acidification in aquatic or terrestrial ecosystems [6]. These N losses are major obstacles to utilizing cattle manure as an organic fertilizer in sustainable and climate-smart agriculture. Consequently, smart and innovative management approaches are prerequisites for reducing environmental pollution [7] and sustaining farmyard manure (FM) as an eco-friendly smart fertilizer [8].

Nanotechnology has recently emerged as a smart technique intended to resolve the problems of modern agriculture [9,10,11]. In materials science, this technology reduces the size of the synthesized material and improves the surface potential, porosity, dispersivity, surface area, and surface functional groups of many modern materials [12]. These properties make them smart materials, which are utilized for the targeted transport of nutrient or pesticides [12,13], improving the nutrient utilization efficiency of conventional or modern fertilizers [9,14] and solving nutrient loss problems in agriculture [15,16]. Therefore, nanotechnology has the potential to synthesize materials that are suitable for reducing environmental losses from conventional fertilizers (organic or synthetic chemicals), such as FM.

Nanobiochar (NB: <100 nm size) can be produced from carbonaceous material through pyrolysis [17,18] and ball milling techniques [19]. NB comprises ultrafine particles, a negative charged surface, reactive organic species, free radicals, and a surface area higher than the size [20]. These properties can increase NB mobility in any media and stability in the colloidal form [21], making it a potential contender or substitute for other nanomaterials for use in agriculture [22]. For instance, NB’s higher surface area than size and negatively charged surface can make it a good adsorbent of nutrients and immobilizing agents of chemicals or hazardous substances in any media, especially in the soil [20]. This nutrient adsorption ability was observed by Chen et al. [23], who found a 14% to 60% reduction in nitrate losses after the application of 0.1% to 1% coconut NB to the soil of the China Loess Plateau. Yang et al. [24] also observed that coconut shells’ NB (0.1% to 1% in mass) reduced the surface transport of N to the deeper soil, increased the potassium (K) content in the soil’s upper layer (10 cm), and consequently augmented the maize grain and total yield. Liu et al. [25] observed that a high NB concentration (1%) strongly adsorbed Cd in the soil and thus reduced its toxicity in *Brassica chinensis* L. Consequently, nanobiochar can act as a strong adsorbent for nutrients and heavy metals [26]. Therefore, nanobiochar could be important in reducing animal manure nutrient losses and improving crop yield [27] when mixed with FM, owing to its unique characteristics [17]. However, to date, no study so far has investigated the influence of NB on nutrient loss and crop nutrient utilization in FM.

On the other hand, metallic nanoparticles are toxic to soil biota and their associated ecosystem services [28,29,30,31]. The physical contact of smaller-sized particles with the cell surface can rupture the cell membrane [32], and the imbalance between oxygen production and buildup (oxidative stress) in the cell caused by reactive oxygen species (ROS) is the main mechanism of metallic nanomaterial toxicity in soil life [33]. Such toxicity indirectly influences the microbial-associated processes of organic matter decay [28], nutrient mineralization [34], and nutrient uptake from applied fertilizers [29,30]. Similar to metallic nanoparticles, Zhao et al. [35] found that a high concentration of nano-graphene oxide (62 mg L^−1^) caused toxicity to algal growth in aquatic habitats. Huang et al. [36] also observed algal growth inhibition in a treatment where a high concentration (600 mg L^−1^) of NB was applied. They explained that high NB concentrations in aquatic environments cause a higher adsorption of nutrients, thereby decreasing their availability for algal growth [36]. Similarly, Zhao et al. [35] reported that NB produced ROS in algal cells, causing oxidative stress and cell damage. Therefore, similar to aquatic organisms, a higher NB concentration might also be toxic to soil life. Consequently, coapplication of NB with FM in the soil can offer a novel approach for reducing its harmful effects on the environment, but may cause toxicity to soil life, its associated functions of nutrient mineralization, and thereby, crop growth. However, to date, no study has investigated the influence of replacing different concentrations of N from FM by NB on NH_3_ emissions, nutrient mineralization, and crop nutrient uptake from the FMNB mixture.

Several studies have used bulk biochar in combination with fertilizer to enhance soil fertility and crop production [37,38,39,40]. For example, the combined use of bulk biochar and animal manure decreased manure C mineralization and increased microbial N compared with biochar or manure alone [39]. Similarly, bulk biochar produced from farmyard manure when coapplied with chemical fertilizer resulted in a 79% higher soil mineral N than the control. Moreover, this combination also enhanced wheat biological and grain yields and N uptake more than biochar alone [40]. In another study by our group, the use of FM biochar alone in the soil increased the microbial biomass N, P, and K mineralization compared with the control (unpublished data). This resulted in a higher sorghum shoot yield and N uptake than the control, signifying the positive influence of coarse/bulk size FM biochar on soil quality and crop productivity. Hence, bulk biochar produced from FM, when applied alone or in combination with chemical or organic fertilizers, improves soil quality and crop productivity [37,38,40].

Keeping in view the positive influence of bulk biochar and NB on soil properties and crop production, the main aim of this study was to explore the replacement of three concentrations (12.5%, 25%, and 50% of N) of FM with NB on NH_3_ emissions, nutrient (N, P, and K) mineralization, and corn nutrient uptake from these fertilizer mixtures when applied in a clay loam soil. We hypothesized that increasing the replacement concentration of NB in FM would lessen NH_3_ emission losses and slow down the release of nutrients, but decrease microbial biomass, nutrients (K, P, and N) mineralization, and these nutrients’ accessibility in the soil for corn productivity and uptake. To examine this, biochar was prepared from FM using a pyrolysis technique and was extensively ball-milled to obtain NB. The treatments, FM at the recommended rate (100 kg N ha^−1^), and FM and NB, where 12.5%, 25%, and 50% N from FM were replaced with NB, were mixed in clay loamy soil-filled pots, and then corn was sown.

## 2. Results

### 2.1. The NH_3_ Concentration in the Air above Pots

**Figure 1:**
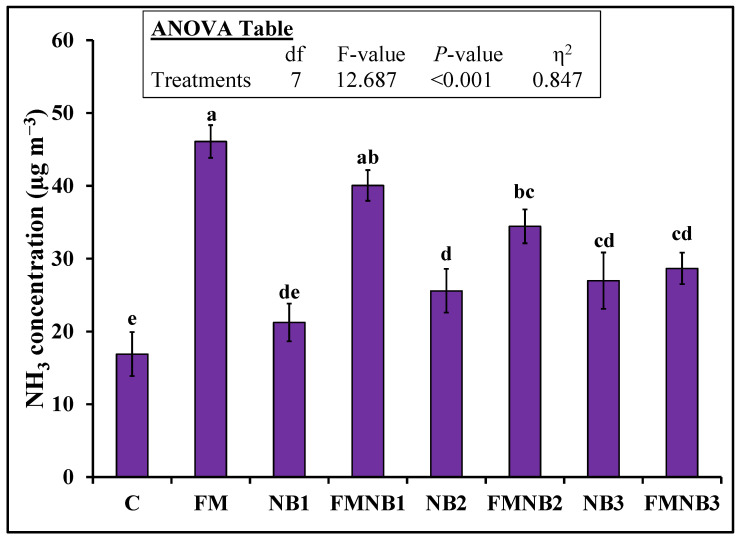
Mean (n = 3) ammonia (NH_3_) concentrations in air 20 cm high from pot soil after individual and coapplication of farmyard manure (FM) with three N replacement dosages (12.5% (FMNB1), 25% (FMNB2), and 50% (FMNB3) of nanobiochar (NB). The control (C) represents untreated soil. Standard errors (±1 SE) among replicates are shown as error bars. Dissimilarity among treatments is represented by different small letters (probability of 5%) after the Tukey-HSD test. The analysis of variance (ANOVA) results are presented in the inset table.

FM application significantly increased the air’s NH_3_ concentration above the pots (Figure 1). The emitted NH_3_ in the FM was 173% (46.1 vs. 16.9 µg m^−3^) greater than that of the control. The application of a lower NB dosage (NB1) alone to the soil did not influence NH_3_ emissions. However, NB2 and NB3 alone increased this concentration by 51% and 60%, respectively, compared to the control. Interestingly, the replacement of 12.5% N (FMNB1) with NB in FM treatments tended to reduce the NH_3_ concentration from the mixture, but this was not statistically significant compared with FM alone (Figure 1). Both FMNB2 and FMNB3 decreased the NH_3_ concentration from the mixture above pots by 25% (34.4 vs. 46.1 µg m^−3^) and 38% (28.7 vs. 46.1 µg m^−3^) than the FM-alone treatment. Nevertheless, no significant change in the NH_3_ concentration above pots was observed between the FMNB2 and FMNB3 treatments (Figure 1).

### 2.2. Influence of Nanobiochar, FM, and Their Mixture on Soil Characteristics

**Figure 2:**
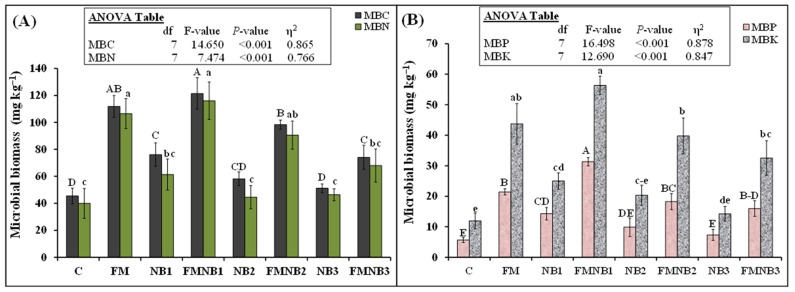
Mean (n = 3) (**A**) microbial biomass carbon (MBC), nitrogen (MBN), (**B**) phosphorous (MBP), and potassium (MBK) at the end of the experiment as affected by individual and coapplied farmyard manure (FM) with various dosages of nanobiochar (NB). Control (C) represents untreated soil. Standard errors (±1 SE) among replicates are shown by error bars. Dissimilarity among treatments is represented by different small (MBN and MBK) and capital letters (MBC and MBK) at a probability of 5% after the Tukey-HSD test. Analysis of variance (ANOVA) outcomes are presented in the inset table.

All treatments significantly influenced nutrient (C, N, P, and K) content in microbial biomass (Figure 2). The NB1 treatment had a 67% (76.1 vs. 45.5 mg kg^−1^) higher MBC than that of the control. However, the NB2 and NB3 treatments did not influence MBC. In the FM treatment, the MBC was 146% (112 vs. 45.5 mg kg^−1^) higher than that of the control. Interestingly, FMNB1 and FMNB2 did not further increase the MBC. However, the FMNB3 mixture decreased 34% (74.1 vs. 111.8 mg kg^−1^) of MBC than FM alone (Figure 2A). Similar to MBC, MBN in the FM treatment was 166% (106.5 vs. 40.0 mg kg^−1^) greater than that of the control. The mixing of NB1 and NB2 in FM did not further influence MBN. However, FMNB3 decreased MBN by 36% (68.0 vs. 106.5 mg kg^−1^) compared with FM alone (Figure 2A). The NB1 application significantly increased MBP and MBK by 152% (14.3 vs. 5.7 mg kg^−1^) and 109% (25.0 vs. 11.9 mg kg^−1^), respectively, than control (Figure 2B). However, treatments with the presence of NB2 and NB3 alone did not affect these parameters. The FM increased MBP and MBK by 278% (21.4 vs. 5.7 mg kg^−1^) and 267% (43.7 vs. 11.9 mg kg^−1^) than control. Similarly, FMNB1 increased MBP by 46% (31.3 vs. 21.4 mg kg^−1^) than FM alone, but this treatment did not influence MBK. Similarly, FMNB2 and FMNB3 did not affect MBP or MBK. Although both parameters tended to decrease in the FMNB2 and FMNB3 treatment groups, these trends were not statistically significant (Figure 2B).

**Figure 3:**
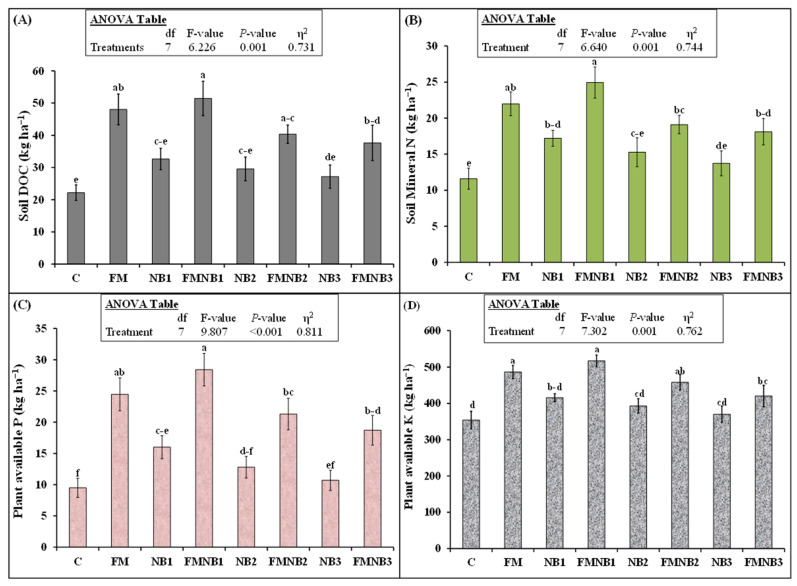
Mean (n = 3) (**A**) soil dissolved organic carbon (DOC), mineral nitrogen (**B**), P (**C**), and K (**D**) at the end of experiment as affected by individual and coapplied farmyard manure (FM) with various dosages of nanobiochar (NB). The control represents untreated soil. Standard errors (±1 SE) among replicates are shown by error bars. Dissimilarity among treatments is represented by different small letters (probability of 5%) after the Tukey-HSD test. Analysis of variance (ANOVA) outcomes are presented in the inset table.

All fertilizer treatments affected the dissolved organic C (DOC) in the soil (Figure 3A). A significant difference in DOC was detected among the NB, FM, and FMNB mixture treatments (Figure 3A). The NB1 treatments did not affect DOC. FM increased this parameter by 117% (48.0 vs. 22.2 kg ha^−1^) compared with the control. A 132% (51.4 vs. 22.2 kg ha^−1^) increment in DOC was detected in FMNB1 compared with the control. FMNB1 increased this parameter by 7%; however, this was not statistically different from FM alone. On the other hand, FMNB3 reduced DOC by 22% (37.6 vs. 48.0 kg ha^−1^) compared with FM alone.

The influence of treatments on soil mineral-N content was significant (*p* = 0.001; Figure 3B). NB1 application significantly enhanced the soil mineral N, which was 48% higher than that of the control. However, the NB2 and NB3 treatments did not influence mineral-N. In contrast with NB2 and NB3, FM treatment increased soil mineral N by 89% (22.0 vs. 11.6 kg ha^−1^) compared with the control. FMNB1 tended to increase mineral-N content compared with FM alone, but the increase was not significant. FMNB2 and FMNB3 tended to decrease mineral-N compared with FM, but the difference was also not significant.

Similar to mineral N, soil P was highest in the FMNB1 treatment (Figure 3C). All FMNB mixture treatments augmented this parameter in the soil compared with the unfertilized control. NB1 increased soil P by 69% (16.0 vs. 9.5 kg ha^−1^) compared with the control. However, NB2 and NB3 alone were not able to increase this parameter more than in the control. FM alone increased this parameter by 157% and FMNB1 increased this by 199% compared with the control. Although plant-available P was 16% higher in the FMNB1 treatment than in FM, the difference was not significant. FMNB2 and FMNB3 tended to decrease soil P but they were not statistically different (Figure 3C).

The treatments significantly influenced the soil K content (Figure 3D). Nanobiochar alone, irrespective of the application rate, did not affect plant-available K in the soil. Again, the greatest increase in K was found in FMNB1, which was 46% (517 vs. 354 kg ha^−1^) greater than that of the control. FM increased this parameter by 37% compared with the control. FMNB1 and FMNB2 did not significantly affect this parameter compared with FM. However, FMNB3 decreased K in the soil by 14% (420 vs. 487 kg ha^−1^) compared with FM (Figure 3D).

### 2.3. Influence of Farmyard Manure, Nanobiochar Alone, and Their Mixtures on Plant Growth Characteristics

Plant height was significantly augmented by all treatments in the control (Table 1). FM increased this parameter by 36% compared with that of the control. None of the FMNB mixtures increased this parameter further. Stem girth was only increased by FMNB1 compared with that in the control. This increment was 31% higher than that of the control. FM application increased the chlorophyll content by 83% compared with the control. A single application of NB1, NB2, and NB3 increased this parameter by 51%, 45%, and 21%, respectively, compared with the control, indicating that increasing the concentration of NB reduced the increase in chlorophyll content (Table 1). FMNB1 increased this parameter by 12%, but FMNB2 and FMNB3 decreased this parameter by 12% and 16%, respectively, compared with FM alone, indicating the negative effect of a high NB dose on leaf chlorophyll content. The FM application increased the leaf area index by 48% over control. Only a single application of NB1 increased this parameter by 29% compared with the control. Other treatments did not significantly affect this parameter (Table 1).

### 2.4. Influence of Farmyard Manure, Nanobiochar Alone, and Their Mixtures on Corn Dry Matter Yield, and Nutrients Uptakes

**Figure 4:**
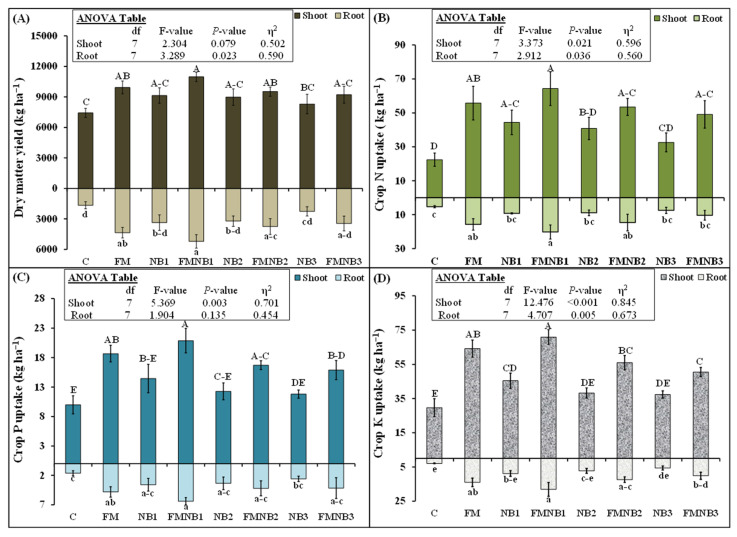
Mean (n = 3) corn plant dry matter (DM) yield, (**A**) N, (**B**) P, (**C**) and K (**D**) uptakes after individual and coapplied farmyard manure (FM) with various dosages of nanobiochar (NB). The control represented untreated soil. Error bars showed standard errors (±1 SE) of the mean. Small letters illustrated significant differences among treatments for root and capital letters for shoot uptakes at a 5% probability level after the Tukey-HSD test. The inset table represented the outcomes of the analysis of variance (ANOVA).

The application of NB alone did not increase corn root or shoot dry matter yield. However, the manure treatments significantly increased this parameter compared with the control, except for FMNB3. Interestingly, irrespective of the dose, the FMNB mixture was not able to further increase the root or shoot DM yield compared with FM alone. FM alone increased shoot and root DM yields by 34% and 162%, respectively, compared with the control. NB1 application alone increased shoot N uptake by 98% more than that of the control, although NB2 and NB3 tended to increase this parameter, but the difference was not statistically significant (Figure 4B). Similarly, FM increased this parameter by 149% compared with that of the control. The addition of any dose of NB in FM did not further affect this parameter. The maximum N uptake by root was present in FMNB1, followed by FM and FMNB2. However, FMNB3 did not increase root N uptake from control. Root N uptake was 197% higher in FM and 281% higher in FMNB1 compared with that of the control. Root and shoot P uptake was the highest in FMNB1, followed by FM and FMNB2 (Figure 4C). Interestingly, NB alone was not able to increase root and shoot P uptake compared with that of the control. Shoot P was 87% and 109% greater in FM and FMNB1 treatments compared with the control (Figure 4C). Similarly, root P uptake in FMNB1 and FM was 293% and 196% larger, respectively, than that of the control. Root P uptake in FMNB3 did not differ significantly from the control. Much like most other uptake parameters, shoot and root K uptakes were also the highest in FMNB1 treatment, followed by FM, FMNB2, and FMNB3. Among NB alone treatments, only NB1 alone was able to increase N uptakes in both root and shoot compared with the control. NB2 and NB3 were not able to increase N uptake. FM increased the shoot K uptake by 116%. NB1 and NB2 mixing in FM was not able to further increase this parameter in shoot compared with FM alone. Remarkably, NB2 and NB3 decreased K uptake in a shoot by 15% (50.5 vs. 64.2 kg ha^−1^) and 27% (56.0 vs. 64.2 kg ha^−1^) compared with that of FM (Figure 4D).

## 3. Discussion

We expected that replacing different concentrations (12.5%, 25%, and 50%) of N by nanobiochar in FM would proportionally decrease NH_3_ emissions from the mixture after its soil application. We observed that all doses tended to decrease NH_3_ emissions, but it was significantly lower in the intermediate and the highest NB doses (Figure 1). The following processes played an important role in reducing NH_3_ emissions when bulk biochar was applied to the soil [41]: (i) the NH_4_^+^ ⇆ NH_3_ equilibrium can be shifted by the biochar pH, (ii) the biological activity in the soil reduces the production of NH_4_^+^ and increases its consumption, (iii) biochar provides more adsorption sites for adsorbing NH_4_^+^ or NH_3_ in the soil. Soil and nanobiochar pH in our study did not differ significantly, but the pH of FM was slightly lower than NB (Table 1), indicating that nanobiochar pH did not play a major role in reducing NH_3_ emissions from the applied FM in NB and FM mixture treatments. Likewise, nanobiochar (pH = 7.7) did not cause an immediate liming effect because of the slightly alkaline nature of our soil (pH = 7.5). Therefore, an increment in microbial NH_4_^+^ consumption, especially under acidic soil conditions as indicated by Ball et al. [42], was ruled out in our study. Moreover, the negative charge surface of NB can absorb NH_4_^+^ ions and decrease their availability. This might happen in our study since the zeta potential (i.e., surface charge) of NB was −26.8 mV. Therefore, in all probabilities, nanobiochar owing to its negative charge surface and more surface area than size may provide more adsorption sites to adsorb NH_4_^+^, NH_3_, or both in our study. These opinions are coherent with Jones et al. [43] who noted that NH_4_^+^ ions were adsorbed on biochar adsorption sites.

The NB surface area provided insight into the adsorption mechanism of NH_4_^+^ [44]. The small size (2.56 Å) [45] makes NH_3_ a suitable candidate to adsorb on biochar surface. This observation was also confirmed by Thangarajan et al. [46] who observed in their study that biochar particles with low surface area (104.68 m^2^ g^−1^) provided more suitable sites for NH_3_ adsorption. Likewise, in a literature review by Ramanayaka et al. [47], it is reported that the surface area of nanobiochar was in the range of 5.6 to 47.2 m^2^ g^−1^, which is much smaller than bulk biochar. Consequently, this greater surface area to size ratio and the surface energy of particles might allow nanobiochar to reduce more NH_3_ emissions in our study (Figure 1).

Our findings are partly in agreement with our hypothesis that replacing FM with NB at 12.5% of N significantly increased microbial biomass P compared with FM alone, but microbial C, N, and K were not affected. Additionally, this NB concentration significantly increased microbial biomass when applied alone to the soil (Figure 2). This showed the positive influence of low NB concentrations on soil microorganisms (Figure 2). Liu et al. [25] explained that the application of NB provided nutrients and other suitable conditions for the favorable growth and activity of microbes. This is in agreement with Zhou et al. [48] who also found that the provision of high nutrient content by nanobiochar increased soil microbial biomass and diversity. According to Chen et al. [49], a finer sized biochar increased microbial phospholipids fatty acids more than medium and coarser sized biochar. They explained this effect by the greater surface area and porous structure of fine-sized particles that enhanced nutrient availability and the protection of microbes against predators. In our study, the increment in microbial biomass might be associated with the accessibility of C and other nutrients in the soil of NB or NB1-mixed FM treatment (Figure 3), which provided a food source for microbial activity and growth. In line with this observation, Montaño et al. [50] also noted that microorganism activity and biomass were higher in those treatments where DOC, mineral N, and other nutrients were high. These food-based relations were also confirmed by Tan et al. [51] who found in their study that microbial communities obtained their food from biochar. Therefore, in all likelihood, the presence of high nutrients and an improved habitat in our study (Figure 3) were responsible for higher increments in the microbial biomass K, N, P, and C in FM and FMNB1 treatments (Figure 2).

In contradiction to our hypothesis, we observed that replacing higher concentrations (25% and 50% of N) of FM with NB tended to significantly decrease all the studied nutrients in microbial biomass (Figure 2), indicating that these concentrations caused toxicity to the soil’s microbial life. According to Dempster et al. [52], an increase in biochar addition rate decreased the microbial biomass C as well as the microbial community-level physiological profile. They explained that biochar rate and type (feedstock and temperature, etc.) might play a role in decreasing microbial biomass C. Sewage sludge derived biochar-added toxic elements from the soil, which decreased the microbial biomass, and this decrease was proportional to the increasing rate of biochar application in the soil [53]. Alternatively, Zhang et al. [54] observed that heavy metals were enriched in cow manure biochar compared with that of cow manure alone. They explained that an increasing pyrolysis temperature from 300–700 °C can increase the stable fraction of heavy metals in cattle manure biochar. In our study, the temperature of the pyrolysis apparatus was fixed at 500 °C; therefore, as observed by Zhang et al. [54], there were fewer chances of heavy metals leaching from the biochar if those were present.

According to Zhang et al. [54], the presence of heavy metals in animal manure is completely reliant on the composition of cattle feed intake. The farmyard manure used in our study was collected from a farm where cattle were grazing on grasses and their feed consisted of farm legumes (*Medicago sativa* and *Trifolium alexandrinum*) and fodder crops (maize, sorghum, and wheat straw etc.) with no concentrate addition. Therefore, we believed that the heavy metals intake in the animal feed was close to zero. Consequently, the presence of toxic elements in the NB or FM would not explain the microbial biomass decrement in our study. Rather, the size of the particle along with the biochar addition rate would be important in the reduction of microbial biomass [49]. Accordingly, Jaafar et al. [55] observed lower microbial biomass C and P where smaller, woody, biochar particles were applied compared with coarser ones, however, this effect was time-dependent. Ideally, the smaller-sized particles possess a high surface area, therefore, these have the potential to attach more microbes on the surface and provide better a microbial habitat than that of coarser ones [55]. However, when NB is applied to the soil, its surface properties may alter due to interaction with soil particles and organic matter; therefore, it might impact microbial attachment to the particle surface [56] and thus influence the microbial biomass. In another recent study, the smaller-sized biochar particles induced positive effects on microbial abundance and biomass, however these particles had less C storage and a higher rate of decay than thicker particles [49]. Consequently, such positive effects would diminish with time when this labile C decomposed in days or months [57,58]. Therefore, as evident from our study, and also concluded in a recent literature review, the adverse effects of smaller-sized biochar on microbial biomass and activity over a longer period should be explored in future studies [59].

The nano-size and surface potential (positive or negative) of the biochar-like metallic nanoparticles might have the capability to disrupt the microbial cell membrane [32]. Accordingly, Kang et al. [60] found that the direct interaction of C nanotubes with bacteria damaged its cell membrane. Moreover, physical cytotoxic characteristics and cell penetration of C-nanotubes and oxidative stress caused by ROS generation were the main mechanisms of C-based nanomaterials toxicity to microorganisms and hence may cause their death [61]. Accordingly, Zhang et al. [62] observed that C-based nanotubes triggered cellular damage in algal cells. Such a process could occur in our study where nano-sized particles might damage the microbial cell membrane due to physical penetration in addition to NB surface potential and would be the main causes of lower microbial biomass in FMNB2- and FMNB3-amended soil (Figure 2).

In contradiction to our hypothesis, we found that increasing the concentration of NB in the fertilizer mixture tended to decrease the nutrients’ (N, DOC, and P) content in the soil, but these effects were statistically non-significant (Figure 3). The reduction in P content might be linked to a decrease in the resupply or diffusion capacity of biochar from the solid part of the soil to the solution [63]. Only the lower concentration of NB in the fertilizer mixture tended to increase these nutrients in the soil, but the increase was also not statistically significant. The high NB concentration in the fertilizer mixture significantly reduced K in the soil compared with FM alone (Figure 3D) and thereby, K uptake by corn crop (Figure 4). This showed that 25% and 50% NB, when replaced with FM, possibly caused toxicity to K, P, N, and C mineralization in the fertilizer mixture. According to Huang et al. [36], the NB increased the nutrient adsorption and thereby decreased their availability for the growth and development of algae. Moreover, they also observed that NB generated ROS in the algal cells and caused oxidative stress to damage these cells. This could be the reason why we observed lower microbial biomass in these treatments (Figure 2), indicating that high concentrations of nanobiochar in fertilizer mixture decreased the biomass and activity of microorganisms. Therefore, this lower presence of microbes leads to lower decomposition and mineralization of organic fertilizer and therefore, a lower availability of K, P, and N nutrients in the soil (Figure 3).

Nutrient accessibility in the soil for crop uptake from organic fertilizers is solely dependent on the presence and activity of soil microorganisms and fauna [64,65,66]. When organic fertilizer is applied to the soil, it undergoes microbial and soil faunal colonization [67,68] that determines the decomposition and nutrient mineralization-immobilization turnover [69] and thereby, nutrient accessibility for crop uptake [70]. The application of high doses of nanomaterial in the soil caused toxicity to the soil microorganisms and fauna [71,72] and thereby reduced the organic amendments’ decomposition and nutrient mineralization and eventually, the crop yield [29,30]. Therefore, the lower nutrient availability in the high concentrations of NB in our study (Figure 3) was well explained by the lower microbial biomass (Figure 2).

Similarly, other activities related to nutrient cycling were also influenced by the application of high doses of nanomaterials in the soil. According to Zhao et al. [73], higher concentrations of (400 mg kg^−1^) carbon nanomaterials decreased different enzymatic (dehydrogenase and phosphatase) activities related to C and N cycling in the soil. In line with this, several other studies also reported that high doses of metallic-based nanoparticles decreased microbial count, their biomass and activity, decay, and nutrient mineralization of organic fertilizers in the soil, which led to a decline in nutrient uptake and the yield of different crops in agroecosystems [28,29,30,34,74]. All these studies proposed that the decline in microbes was the main mechanism of decreasing decay and nutrient mineralization of organic fertilizer by nanoparticles. Hence, in line with these studies, we also proposed that a decline in microbial biomass (Figure 2) and activity would lead to a decrease in the decomposition and nutrient mineralization of high concentrations of nanobiochar and FM mixture (Figure 3) treatment. Hence, we observed a decrease in K or a tendency to decline in N and P uptakes by corn root and shoot (Figure 4).

## 4. Materials and Methods

### 4.1. Nanobiochar Production

We collected the fresh FM from a buffalo farm situated in Rawalpindi, Pakistan. This manure was dried under the shade in an open place. The dried manure was used to produce biochar at 500 °C for 5 h in a conventional pyrolysis tank [40]. Nanobiochar was prepared with ball milling and sieving techniques [19,75]. Using these methods, the bulk biochar was placed in the tank of the ball mill (F-P4000, Huanyu Instrument, Zhejiang, China). The milling technique was used to extensively grind the bulk biochar at 300 rpm for 24 h. In this technique, the biochar was crushed by 3 mm stainless steel balls (n = 800) present in a stainless-steel tank (volume 500 mL) and laterally, the crushed biochar was sieved through a 200-mesh screen to get NB.

### 4.2. The Characteristics of Nanobiochar

**Figure 5:**
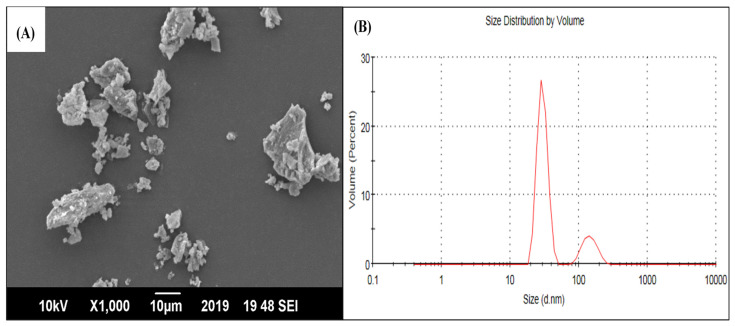
The image of scanning electron microscope showing dispersed nanobiochar with an angular blocky structure (**A**) and Zeta sizer analysis (**B**).

The nanobiochar surface charge and size were analyzed by a Zeta sizer (Nano ZS, Malvern Instruments, Zeta-PALS, UK). For this purpose, an aqueous suspension of NB was prepared and subjected to the zeta-potential analyzer (Malvern Instruments, Zeta-PALS, UK). Surface morphological analysis of NB was carried out by SEM (S-4700, Hitachi, Japan). Using the sputter deposition technique, gold particles were sputtered on the NB surface through a sputtering apparatus (JEOL JFC-1500, Kyoto, Japan). These sputtered samples were subjected to particle surface morphological analysis at 20 Kv and a 25–50,000× magnification range in SEM. The SEM image showed the flakey/crumbly nature of NB particles displaying polygonal shapes (Figure 5A). Their agglomeration makes it difficult to determine the exact size of each NB particle. However, their estimated average size was <1 μm. This agglomeration nature could be explained by the existence of static charges on the NB surface. According to the zeta sizer, the NB particles’ size ranged between 50 and 490 nm (Figure 5B).

### 4.3. Pot Experiment

A standard pot trial was started in mid-March 2020 in the designated area for research (32.9303° N latitude and 72.8556° E longitude with 2500 feet altitude from sea) in close vicinity to the Agronomy Department, PMAS-AAUR, Rawalpindi, Pakistan. The air temperature of the area ranged between 8 and 32 °C and rainfall ranged from 0 to 235.6 mm.

The clay loam soil was obtained from the PMAS-AAUR research farm. The government of Pakistan classified this soil as Rawal series, Udic Haplustalf Alfisols. To remove the stone and root fragments, the soil was sieved with 2 mm mesh. Then, thirteen kilograms of soil was added in one pot with a diameter of 23.5 cm and an area of 0.043352 m^2^. The initial chemical compositions of various nutrients observed in the soil, FM and NB, are presented in Table 2. Based on these compositions, different concentrations/dosages of NB were calculated from the recommended N application rate of the corn crop. The recommended rate for N in corn is 100 kg N ha^−1^ to get optimum yield. Normally, N is applied in two doses if chemical fertilizer is used. Since organic fertilizer was utilized in this experiment, total N was applied in the soil as a basal dose at the start of the experiment. In total, eight treatments were used: (i) unfertilized control (C), (ii) FYM at recommended N rate (FM; 100 kg N ha^−1^) for corn crop, (iii) nanobiochar alone applied at 12.5% of the recommended N rate (NB1), (iv) nanobiochar alone, 25% of N recommendation (NB2), (v) nanobiochar alone, 50% of N recommendation (NB3), (vi) a mixture of FM (87.5%) + NB1 (12.5%), (FMNB1), (vii) a mixture of FM (75%) + NB2 (25%) (FMNB2) and (viii) a mixture of FM (50%) + NB3 (50%), (FMNB3). The 12.5% of nanobiochar treatment added 12.5 kg of N, 6.25 kg of P, and 18.75 kg ha^−1^ of K to the soil. Similarly, the respective addition of K, P, and N quantities in the soil were 37.5, 12.5, and 25 kg ha^−1^ by the second NB dose whereas the final treatment (where 50% N from farmyard manure was replaced with nanobiochar) added 75%, 25%, and 50% of K, P and N, respectively, to the soil through nanobiochar. Corn cv. Gorilla-F1 seed was purchased from the local market and six of these seeds were used to sow corn in a single pot. Following germination, some of the corn plants were removed to maintain 3 plants per pot. Each treatment consisted of 3 replications. All these pots were placed according to a complete randomized design (CRD) under an open space to offer natural environmental conditions. Soil moisture was retained at 60% WHC in each pot by monitoring it with a moisture meter (FY-901, Hangzhou, China). The difference in moisture content was maintained by regularly irrigating pots with a hand sprinkler to avoid any drought stress during the experimental period.

### 4.4. Measurement of Ammonia Emission

A parallel pot experiment with the same treatments was set up next to the abovementioned trial to measure ammonia concentrations in the air above the pots. Immediately after treatment application to the pots, three passive flux samplers were mounted vertically in a wooden piece above each pot to capture ammonia [76]. In the pot center, each wooden piece had samplers fixed in the vertical direction and the open side of the sampler towards a downward direction was installed at a 20 cm height from the surface of the soil for 72 h. A 15 m distance was maintained amidst two adjacent pots to elude NH_3_ mix-up among the experimental pots [77]. Each sampler consisted of steel grids where 60 μL H_2_SO_4_ (10% (*w*/*v*)) was coated to capture NH_3_. After day 3, samplers were detached from a wooden frame and a lid was used to immediately close their opening. These samplers were put in the refrigerator at 4 °C until the next examination [78]. To analyze the NH_4_^+^-N content, each steel grid was removed from the sampler and washed with 5 ml of deionized water. Afterward, NH_4_^+^-N in the solution was examined [78] as discussed in Hofschreuder et al. [79]. Equations (1) and (2) were used to determine the mean NH_3_ (μg m^−3^) concentration:

(1) $$C_{n}{NH}_{3}=\frac{A{NH}_{4}^{+}\times {Ll}_{t}}{{dC}_{o}\times A_{r}t\times T}\times \frac{17}{18}$$

(2) $${dC}_{o}=\frac{Te\;1.75}{Pre}\left(1.1265\times 10-9\right)$$

where C_n_NH_3_ signifies NH_3_ (μg m^−3^) concentration, ANH_4_^+^ denotes NH_4_^+^-N concentration (mg) detected in deionized water, and Ll_t_ shows the length of the tube (0.041 m). dC_o_ designates a coefficient of diffusion with the value 0.0000247 m^2^/s/day used for this procedure. A_r_t expresses inner tube area (0.0000785 m^2^), T is sampling time (seconds), Te denotes the temperature of air (Kelvin, 302.81), and Pre indicates air pressure (1.0023 bar).

### 4.5. Microbial Biomass

The fresh soil was taken to determine microbial biomass C (MBC) and N (MBN) following fumigation and then, the extraction method. For this analysis, 10 g of fresh soil was taken and divided into two equal portions. One half was used to fumigate with 25 mL of CHCl_3_ (ethanol-free) in a vacuum desiccator for 36 h at room temperature. Afterward, CHCl_3_ fumes in the soil were exhaled by putting the fumigated soil into a hot water bath for 2 h where the temperature was set at 80 °C. Subsequently, the remaining half of the fresh soil was transferred into plastic vials where 25 mL K_2_SO_4_ solution (0.5 M) was mixed with a reciprocal shaker (250 rpm) for 30 min. The solutions were then filtered, and resultant extracts were analyzed for total C through the TOC apparatus (TNM1: Shimadzu, Kyoto, Japan) and total N through the Kjeldahl digestion procedure. The below equation was used for calculating the MBN and MBC:

(3) $$MBN\;or\;MBC=\frac{{TC}_{fu}\;or\;{TN}_{fu}-{TC}_{nfu}\;or\;{TN}_{nfu}}{kECor\;kEN}$$

where TC_fu_, TC_nfu,_ TN_fu_, and TN_nfum_ are the concentrations of total C and N in fumigated and fresh soils, respectively. The coefficient for calculating MBC is kEC (0.45) [80] and for MBN is kEN (0.54) [81].

Microbial biomass P (MBP) was examined using the procedure of Brookes et al. [82]. Microbial biomass potassium (MBK) was also assessed by the above fumigation-extraction technique after some changes; for this purpose, the extraction of P and K was carried out through 50 mL ammonium acetate (C₂H₇NO₂; 1 M) solution [83]. The extracted K content was assessed with a flame photometer (Jenway, PFP7, Thermo Fisher Scientific, Waltham, MA, USA) and P was determined through a spectrophotometer (Shimadzu, UV1800, Kyoto, Japan).

### 4.6. Chemical Properties of Soil and Nanobiochar

The soil was sampled from 0–15 cm deep layers at three random places of each pot before crop sowing and at the final crop harvest. A composite soil sample was formed by mixing these samples. A suspension of the soil to water ratio (1:2.5) was prepared by mixing it for 1 h, then leaving it for 30 min alone to homogenize. An AB-DTPA method was employed to measure mineral N present in the soil [8]. For this purpose, we took 10 g of soil from a composite sample and extracted it with 20 mL of ammonium bicarbonate (1 M) and DTPA (0.005 M) solutions. The extract was analyzed with ion chromatography (Thermo Dionex Integrion HPIC system, Thermo Fisher Scientific, Waltham, MA, USA). For P and K determination, 1 g soil was extracted with CaCl_2_ (0.01 M) solution [84] and the extract was subjected to a spectrophotometer (Shimadzu, UV1800) and a flame photometer (Jenway, PFP7, Thermo Fisher Scientific, Waltham, MA, USA). Wet oxidation was carried out to measure the C in biochar/soil [85]. Dissolved organic carbon (DOC) in the soil was analyzed by putting a 5 g sample in a 50 mL polytube and then adding 25 mL water to make a suspension. This suspension was incubated at 80 °C for one day and DOC was measured using a TOC analyzing apparatus (TOC-VCPH, Shimadzu, Kyoto, Japan) [86].

### 4.7. Plant Growth Attributes

The corn crop was harvested in the first week of June 2020 at its physiological maturity stage after 85 days of crop sowing. Before plant harvesting, shoot height was measured with a meter rod, leaf area meter was used for calculating leaf area, stem girth was measured through a Vernier caliper, and chlorophyll content was determined using a SPAD meter. The fresh corn was weighed up after drying the samples in an oven at 105 °C. DM yield was measured using a digital weighing balance. The corn roots from the pots were manually removed. For this purpose, the entire soil clump was soaked in a water-filled bucket and then the clump was divided into different parts. These parts were positioned on 0.5 mm mesh and soil particles around the root were removed via a tap-water jet. Roots of corn were oven-dried for 2 days at 105 °C and DM content was determined using a digital weighing balance through the weight difference method. From the dried samples, DM yield, K, N, and P content in both the root and shoot were determined. Plant samples were digested with concentrated H_2_SO_4_ (95–97%) and the filtrate was used for N content determination using a Kjeldahl apparatus whereas P and K were digested with concentrated HNO_3_ (70%) and perchloric acid (60%) and the digestate was subjected to instrumental analysis as outlined in Section 4.6.

### 4.8. Corn Nitrogen, Phosphorous, and Potassium Uptakes

The corn nutrients’ uptakes from the applied treatments were calculated using the formulae given below:

(4) $$Nutrient\;uptakes\;in\;control=(N_{m0}\;or\;P_{m0}\;or\;K_{m0}\times {DM}_{m0})$$

(5) $$Nutrient\;uptakes\;in\;fertilized\;treatments=(N_{ma}\;or\;P_{ma}\;or\;K_{ma}\times {DM}_{ma})$$

where N_ma_, P_ma_, and K_ma_ indicate total N, P, and K content in the corn root or shoot of fertilized treatments (kg P, K, and N). DMma signifies DM yield (kg ha^−1^) of the corresponding corn treatment. K_m0_, P_m0_, and N_m0_ denote K, P, and N content detected in the control treatment. DM_m0_ shows corn DM yield in the control treatment (kg ha^−1^).

### 4.9. Statistical Analysis

Treatment influence on soil P, DOC, K, mineral N, and crop DM yield, K, P, and N uptakes from the applied treatments were statistically tested through univariate analysis in the statistical package for social studies (SPSS 20, IBM, NY, USA). The data normalization was examined by Kolmogorov–Smirnov and Shapiro–Wilk tests, and if necessary, data were normalized using SPSS. After meeting the assumptions, analysis of variance (ANOVA) was employed to analyze the main effect of treatments at a probability level of 5%. When the main effects were significant, the means of all treatments were analyzed by Tukey’s honest test.

## 5. Conclusions

This is the first study showing the dose-dependent nanobiochar toxicity to soil microbial biomass, N, P, K mineralization, and corn nutrient uptake from farmyard manure. We observed that a low concentration of NB (12.5% of N) significantly improved microbial biomass when applied alone and there was a tendency toward an increase in N, P, and K minerals and corn root and shoot K, P, and N uptakes when this dose was mixed with FM. This indicated the positive impact of NB (low dose) on soil microorganisms and their activity, nutrient mineralization, and corn nutrients’ uptake from this fertilizer mixture. The highest NB concentration significantly decreased microbial biomass N and C, soil K, and corn K uptake from the fertilizer mixture, causing toxicity to soil microbes and their associated ecosystem services. These findings indicate that NB’s influence on soil microbial biomass, nutrient availability, and their uptake by corn crop is concentration- and nutrient-dependent. These effects could be attributed to the distinctive characteristics (smaller size, high surface area, negative surface, etc.) of NB that could influence soil microbes and their associated processes in the soil differently when applied at different concentrations. Hence, the use of nanobiochar in the agroecosystem would be recommended carefully for improving the quality of soil and crop production. However, detailed, long-term, toxicological mechanistic studies are required with the addition of more soil types and organic fertilizers for assessing the NB toxicity of agroecosystems and to devise the recommendation of NB use in agricultural systems.

## Figures and Tables

**Table 1:** Effect of treatments on plant growth parameters. The treatments are 12.5% (NB1), 25% (NB2), and 50% (NB3) of N from nanobiochar (NB), farmyard manure (FM) alone and their coapplication with FM. The control (C) represents untreated soil. Small letters illustrated significant differences among treatments at a 5% probability level after the Tukey-HSD test. * indicates leaf area index.

Treatment	Plant Height	Stem Girth	Chlorophyll	* LAI
	(cm)	(cm)	(SPAD)	(m^2^/m^2^)
C	80.06 ± 2.42 ^d^	0.81 ± 0.01 ^b^	26.82 ± 1.35 ^e^	3.87 ± 0.37 ^d^
FM	108.61 ± 5.69 ^ab^	0.88± 0.07 ^ab^	49.07 ± 2.43 ^b^	5.75 ± 0.43 ^ab^
NB1	98.44 ± 1.82 ^bc^	0.84 ± 0.07 ^ab^	40.53 ± 1.38 ^c^	5.01 ± 0.21 ^bc^
FMNB1	115.89 ± 7.27 ^a^	1.06 ± 0.01 ^a^	55.00 ± 1.35 ^a^	6.37 ± 0.19 ^a^
NB2	96.17 ± 2.66 ^c^	0.86 ± 0.05 ^ab^	38.89 ± 0.87 ^c^	4.95 ± 0.15 ^b–d^
FMNB2	103.50 ± 4.55 ^bc^	0.98 ± 0.04 ^ab^	42.94 ± 1.05 ^c^	5.50 ± 0.37 ^a–c^
NB3	92.56 ± 2.41 ^c^	0.86 ± 0.05 ^ab^	32.54 ± 1.81 ^d^	4.62 ± 0.72 ^cd^
FMNB3	100.17 ± 2.24 ^bc^	0.89 ± 0.07 ^ab^	41.14 ± 1.43 ^c^	5.25 ± 0.10 ^a–c^

**Table 2:** Mean (n = 3) chemical composition of initial soil, farmyard manure, and nanobiochar used in the study.

Parameters	Units	Soil	Farmyard Manure	nanoB
pH		7.54 ± 0.05	6.50 ± 0.09	7.70 ± 0.21
EC	uS cm^−1^	26.46 ± 1.81	24.03 ± 0.09	29.50 ± 0.46
Total N	%	0.49 ± 0.21	0.90 ± 0.53	0.80 ± 0.06
Total P	%	0.25 ± 0.03	0.60 ± 0.91	0.40 ± 0.01
Total K	%	0.30 ± 0.02	0.90 ± 0.82	1.20 ± 0.10
Total C	%	0.18 ± 0.07	23.2 ± 0.64	51.0 ± 0.88
C:N ratio		36.8 ± 0.42	25.7 ± 0.34	67.5 ± 5.35

## Data Availability

All the data is presented in the manuscript.
